# Enlarged housing space and increased spatial complexity enhance hippocampal neurogenesis but do not increase physical activity in mice

**DOI:** 10.3389/fspor.2023.1203260

**Published:** 2023-09-26

**Authors:** Daisuke Funabashi, Ryuki Tsuchida, Takashi Matsui, Ichiro Kita, Takeshi Nishijima

**Affiliations:** ^1^ Department of Health Promotion Sciences, Graduate School of Human Health Sciences, Tokyo Metropolitan University, Tokyo, Japan; ^2^ Exercise Biochemistry & Sport Neurobiology Division, Institute of Health and Sport Sciences, University of Tsukuba, Tsukuba, Japan

**Keywords:** physical activity, environmental enrichment, housing space, spatial complexity, hippocampal neurogenesis

## Abstract

**Introduction:**

Environmental enrichment (EE) improves various health outcomes, such as hippocampal neurogenesis, in rodents, which is thought to be caused, in part, by increased physical activity. However, the specific effect of each enrichment component, such as enlarged housing spaces and increased spatial complexity with a variety of objects, on physical activity remains unclear because of methodological limitations in measuring physical activity. We aimed to examine whether enlarged housing spaces and increased spatial complexity increase physical activity in mice using a body-implantable actimeter.

**Methods:**

Adult male C57BL/6J mice were assigned to either standard housing or EE groups. The housing environment in the EE mice was gradually enriched by enlarging the housing space and the placement of a variety of objects. Physical activity was measured using a body-implanted actimeter. Hippocampal neurogenesis was immunohistochemically examined.

**Results:**

Enlarged housing spaces and the placement of a variety of objects did not increase physical activity in mice. In contrast, hippocampal neurogenesis was enhanced in the EE mice, suggesting that environmental interventions successfully provided enriched housing conditions for these mice.

**Conclusions:**

These results indicate that enlarged housing spaces and increased spatial complexity do not increase physical activity in mice. Furthermore, we found that EE enhanced hippocampal neurogenesis without increasing activity volume. Besides the current understanding that increasing the amount of physical activity is key to improving hippocampal function, our result suggests that the environment in which physical activity takes place is also a crucial contextual factor in determining the impact of physical activity on hippocampal function.

## 1. Introduction

Despite increasing evidence showing the benefits of physical activity on physiological and mental health (1–3), a large proportion of the global population is physically inactive (4, 5). Although exercise is one of the most effective strategies to promote physical activity, some people struggle to create opportunities for it because of occupational, family, or social circumstances. Therefore, promoting physical activity via environmental or lifestyle changes, independent of encouraging exercise, is required (6). However, an understanding of the environmental factors affecting physical activity levels is still lacking.

In animal studies, environmental enrichment (EE) is a model that enhances physical, sensory, cognitive, and social stimulation and has been proven to have abundant health benefits in rodents (7, 8). EE consists of exercise-associated equipment, such as a voluntary running wheel, many cage mates, an enlarged housing space, and the placement of a variety of objects. In particular, the voluntary running wheel is a potent factor to increase physical activity and largely contributes to several health benefits induced by EE (9). However, it has been demonstrated that EE without a running wheel similarly improves several health outcomes, such as spatial memory, anxiety-like behavior, and hippocampal neurogenesis in rodents (10–12). However, no study has examined whether EE consisting of an enlarged housing space and a variety of objects, but excluding a running wheel, increases physical activity in rodents. This is because previous methods to measure physical activity, including infrared, telemetry, and video-tracking systems, have made it difficult to assess physical activity accurately in rodents housed in large cages and complex environments containing a variety of objects (13).

In our previous study (14), we measured physical activity in mice using a body-implantable actimeter, a recently developed device used to measure physical activity in many species, including rodents. Activity data were recorded independently and did not require other devices, which enabled us to assess physical activity in group-housed rodents under any experimental condition, including a large cage and a complex environment with a variety of objects. The validity of the body-implantable actimeter was confirmed by demonstrating that the number of activity counts measured was highly correlated with those counted by an infrared beam brake system (14). Hence, we aimed to examine whether enlarged housing spaces and increased spatial complexity due to the placement of a variety of objects increase physical activity in mice, using this actimeter.

To assess the specific effect of each environmental component, we employed an enrichment procedure in which the housing space was first gradually enlarged, and then a variety of objects were placed in the cage. In this study, the running wheel was excluded because it is known to be a potent trigger of increased physical activity. Furthermore, based on previous evidence showing that EE enhances brain functions, such as hippocampal neurogenesis (15), hippocampal neurogenesis was also assessed to confirm whether our environmental intervention provided sufficiently enriched housing conditions for mice.

## 2. Method

### 2.1. Animals

Four-week-old male C57BL/6J mice (*n* = 12) were obtained from Japan SLC, Inc. (Shizuoka, Japan) and housed under controlled temperature (22–24°C) and light conditions (12:12-h light and dark cycle, light at 4:00–16:00). Food and water were provided *ad libitum*. All experimental procedures were approved by the Animal Experimental Ethics Committee of Tokyo Metropolitan University.

### 2.2. Experimental design

All the mice were housed in groups (four mice/cage) throughout the experiment. When the mice were 10 weeks old, an actimeter was intraperitoneally implanted to measure their physical activity. One week after surgery, the mice were randomly assigned to either the standard housing (SH, *n* = 4) or EE (*n* = 8) group.

**Figure 1:**
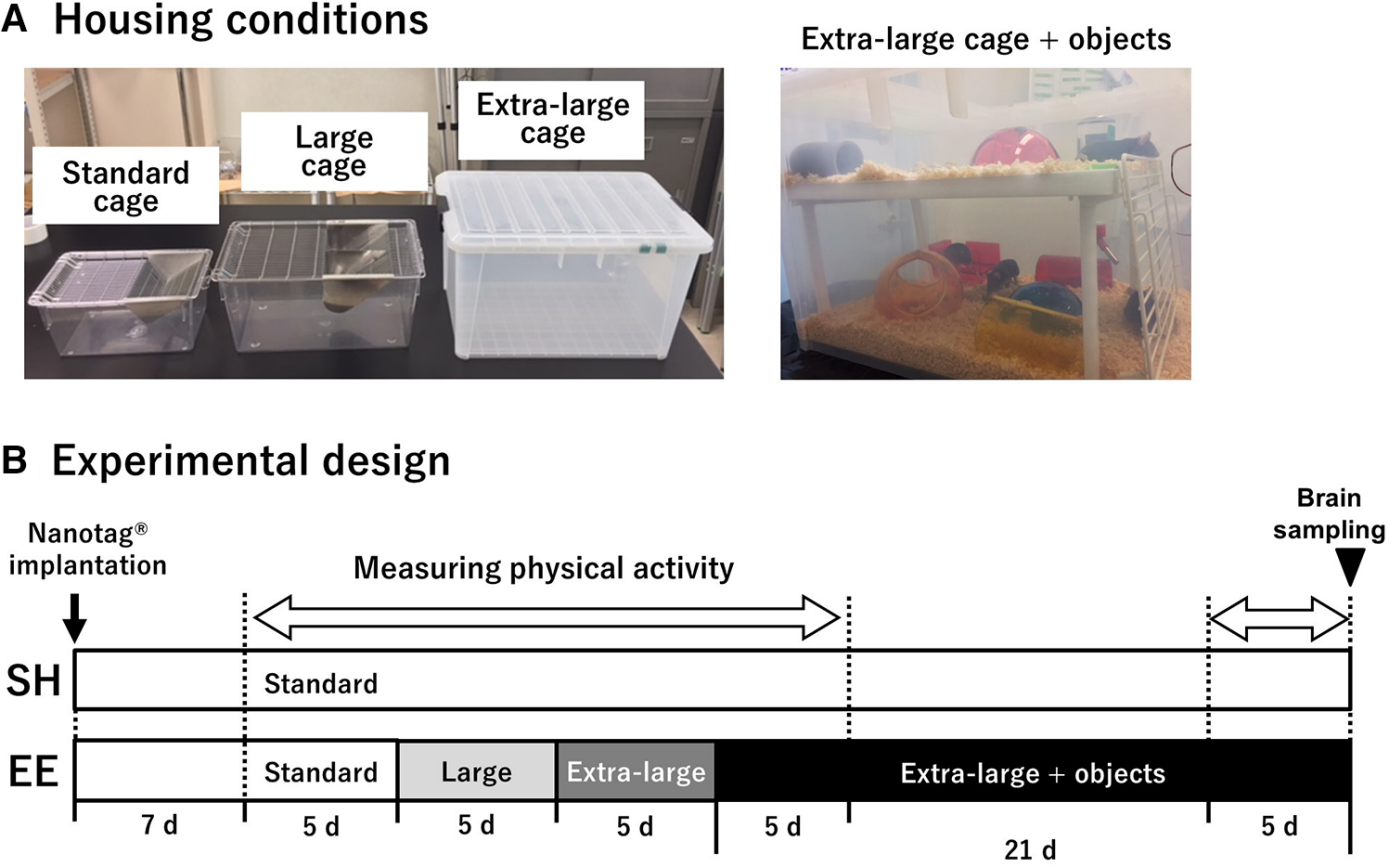
Housing conditions used in the current study (**A**), and experimental design (**B**). A body-implantable actimeter to measure physical activity, nanotag®, was implanted intraperitoneally 1 week before measuring physical activity in mice of both groups. SH mice were housed in standard cages throughout the experiment, whereas EE mice were housed in standard, large, and extra-large cages for 5 days, followed by an extra-large cage with two floors and a variety of objects for 3 weeks. The period of physical activity measurement is indicated by a blank white arrow. At the end of the experiment, the brains of both the groups were removed.

SH mice were housed in standard cages (L × W × H = 225 × 338 × 140 mm; Figure 1A) throughout the experiment. EE mice were housed in standard, large (276 × 445 × 204 mm), and extra-large (370 × 530 × 300 mm) cages for five days, followed by an extra-large cage with two floors and a variety of objects for three weeks (Figure 1B). Any novelty effect accompanied by environmental changes should be eliminated to clarify the effect of each environmental challenges on physical activity. Because a previous study demonstrated that the effect of environmental novelty on spontaneous physical activity was eliminated by 24-hour acclimation (13), we set the period of each different-sized environment for five days.

We used various shapes, sizes, and materials of objects such as igloos, crawl balls, domes, tunnels, and an upper floor with stairs. The objects were changed twice a week in random combinations to provide various sensory stimulations and opportunities for spatial learning. Although mice were not able to perform the hanging activity on the ceiling of the EE cage because the roof was a plastic cover with holes for air ventilation, we observed the use of the upper floor and stairs for this activity. After the experimental endpoint, mice were sacrificed for immunohistochemical examination.

### 2.3. Measurements of physical activity

Physical activity was measured using the body-implantable actimeter, nanotag® (Kissei Comtec Ltd., Nagano, Japan), as previously described (14). At 10 weeks of age, mice were implanted with the nanotag® intraperitoneally under anesthesia via the intraperitoneal injection of an anesthetic cocktail (hydrochloric acid medetomidine, 0.3 mg/kg; midazolam, 4 mg/kg; butorphanol tartrate, 5 mg/kg) (16). After surgery, the mice were intraperitoneally administered atipamezole hydrochloride (an antagonist of medetomidine hydrochloride, 0.3 mg/kg) to recover from the anesthesia, thereby preventing a sustained decline in body temperature. The mice underwent a 1-week recovery period after nanotag implantation because the negative effect on physical activity thereof has been confirmed to be nearly negligible after this period (14).

The nanotag® contains a 3-axis accelerometer and can count both horizontal (x- and y-axes) and also vertical (z-axis) activities. Its internal battery lasts for up to 2 months, and data can be recorded continuously or intermittently for 60 days by turning the power on/off externally. To conserve battery, we stopped and started measuring physical activity during the middle of the 3-week period in an extra-large cage with a variety of objects according to the experimental design (Figure 1B). To start and stop data recording, we touched the radio frequency identification card reader (PaSoRi®) on the nanotag® implanted in the mice.

After the measurements, the stored data in the nanotag® was retrieved and sent to our computer using the PaSoRi®. Data from two mice in the EE group were excluded because their nanotags failed to restart recording.

### 2.4. Immunohistochemical staining

At the end of the experiment, the mice were sacrificed by transcardial perfusion with cold saline while they were deeply anesthetized with pentobarbital sodium (100 mg/kg body weight). The brains were quickly removed and post-fixed in 4% paraformaldehyde in 0.1 M phosphate-buffered saline (PBS, pH 7.4) for 48 h. The brains were cryoprotected in 30% sucrose in PBS and frozen until sectioning using a freezing microtome (REM-710; Yamato Kohki Industrial, Saitama, Japan). On sectioning, we obtained coronal sections (40 µm) to encompass the whole hippocampus and stored these in PBS containing 0.01% sodium azide. The sections were randomly coded, and the investigator was blind to the experimental group for unbiased processing and analysis.

Immunohistochemistry for Ki-67 and doublecortin (DCX) was performed as previously described (17). Free-floating sections were first preincubated with 1% H_2_O_2_ in 0.1 M PBS to quench endogenous peroxidase activity. They were then rinsed in PBS with 0.5% Triton X-100 and 0.5% bovine serum albumin (PBT-BSA), and incubated with rabbit monoclonal anti-Ki-67 antibody (1:1,000, #ab16667, Abcam) and goat polyclonal anti-DCX antibody (1:500, #sc-8066, Santa Cruz Biotechnology) diluted in PBT-BSA for 48 h at 4°C. The sections were then incubated with an appropriate biotinylated secondary antibody (anti-rabbit IgG, #AP182B; anti-goat IgG, #AP180B, EMD Millipore) diluted in PBT-BSA (1:1,000) for 24 h at 4°C. The sections were then treated with an avidin-biotin-peroxidase complex (Vectastain ABC peroxidase kit, Vector Laboratories) for 2.5 h. Finally, the antigens were visualized with 0.02% 3,3-diaminobenzidine in 0.1 M Tris-HCl (pH 7.6) containing 0.001% H_2_O_2_. For Ki-67 staining, the reaction was intensified using nickel ammonium sulfate. Cell nuclei were counterstained with Nissl. The sections were mounted on gelatin-coated slides, dehydrated in a graded ethanol series, and cleared in xylene. The coverslips were then applied.

### 2.5. Assessment of hippocampal neurogenesis

Using eight to ten Nissl-stained sections per mouse, the total volume of the granule cell layer (GCL) in the dentate gyrus (DG) was estimated using the Cavalieri method as described previously (17). To calculate the density of Ki-67-positive cells in the DG, Ki-67-positive cells were manually counted under an optical microscope (×20 objective lens, BX-53, Olympus), and divided by the GCL volume, as determined from an adjacent Nissl-stained section, and averaged over six to eight sections per mouse.

DCX-positive immature neurons in the DG were quantified as described previously (17, 18). DCX-positive immature neurons in the 40-µm-thick sections overlap; as a result, it is difficult to accurately count the number of DCX-positive somas. With reference to previous studies (17, 18), a segmented line was drawn along the middle of the GCL in the DG using ImageJ software (National Institutes of Health, Bethesda, MD), and crossings over the dendrites of DCX-positive immature neurons were counted.

### 2.6. Statistics

Physical activity was analyzed using two-way repeated-measures analysis of variance (ANOVA). If a significant interaction or main effect was observed, Tukey's post-hoc test was used to assess the statistical differences between groups at each time point. Hippocampal neurogenesis results were analyzed using a two-tailed unpaired *t*-test. All data were presented as the means ± standard errors (SEMs). The threshold for statistical significance was set to *P* < 0.05.

## 3. Results

### 3.1. Physical activity

**Figure 2:**
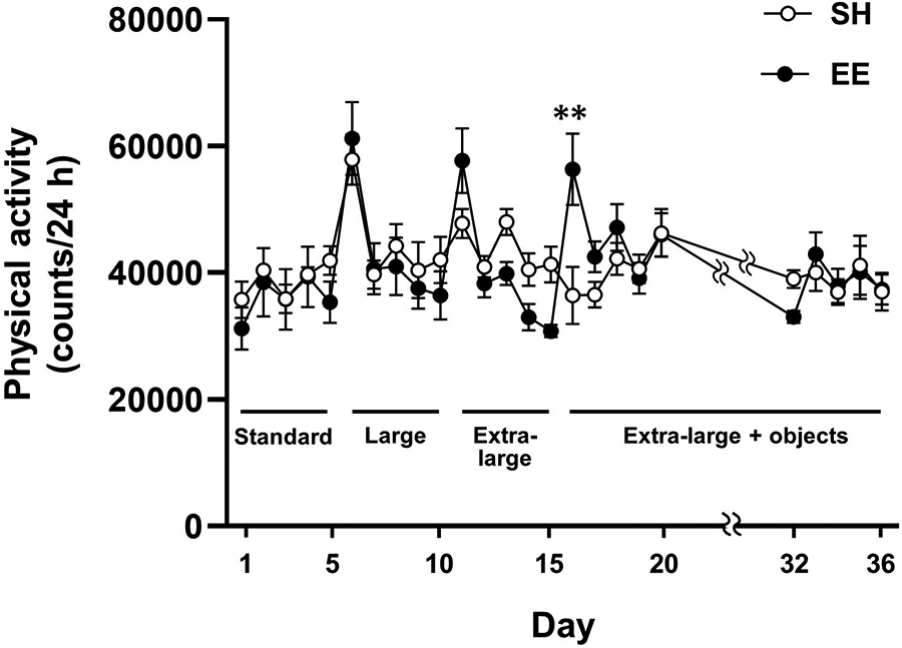
Housing conditions in EE mice changed according to the experimental design (figure 1B), and SH mice were housed in the standard cage throughout the experiment. **P < 0.01 between groups at each time point. Values represent means ± SEM (*n* = 4–6 per group).

To examine whether enlarged housing spaces and enhanced spatial complexity increased physical activity in mice, we compared the physical activity between the SH and EE groups (Figure 2). Two-way repeated-measures ANOVA showed a significant main effect of time [F(24, 192) = 11.16, *P* < 0.0001], whereas no main effect of group [F(1, 8) = 0.0191, *P* = 0.894] was observed. A significant group × time interaction was observed [F(24, 192) = 3.528, *P* < 0.0001], and we compared physical activity between the groups at each time point. Following a post-hoc analysis, physical activity was higher in the EE mice than in the SH mice on the first day in the extra-large cages with a variety of objects (*P* < 0.01). Additionally, we observed increased physical activity in the EE mice on the first day under each housing condition, likely as a result of increased exploratory behavior induced by a novel environment. Considering that physical activity did not differ between the SH and EE mice, except on the first day in extra-large cages with a variety of objects, enlarged housing space and increased spatial complexity may not be capable of increasing physical activity in mice.

### 3.2. Hippocampal neurogenesis

**Figure 3:**
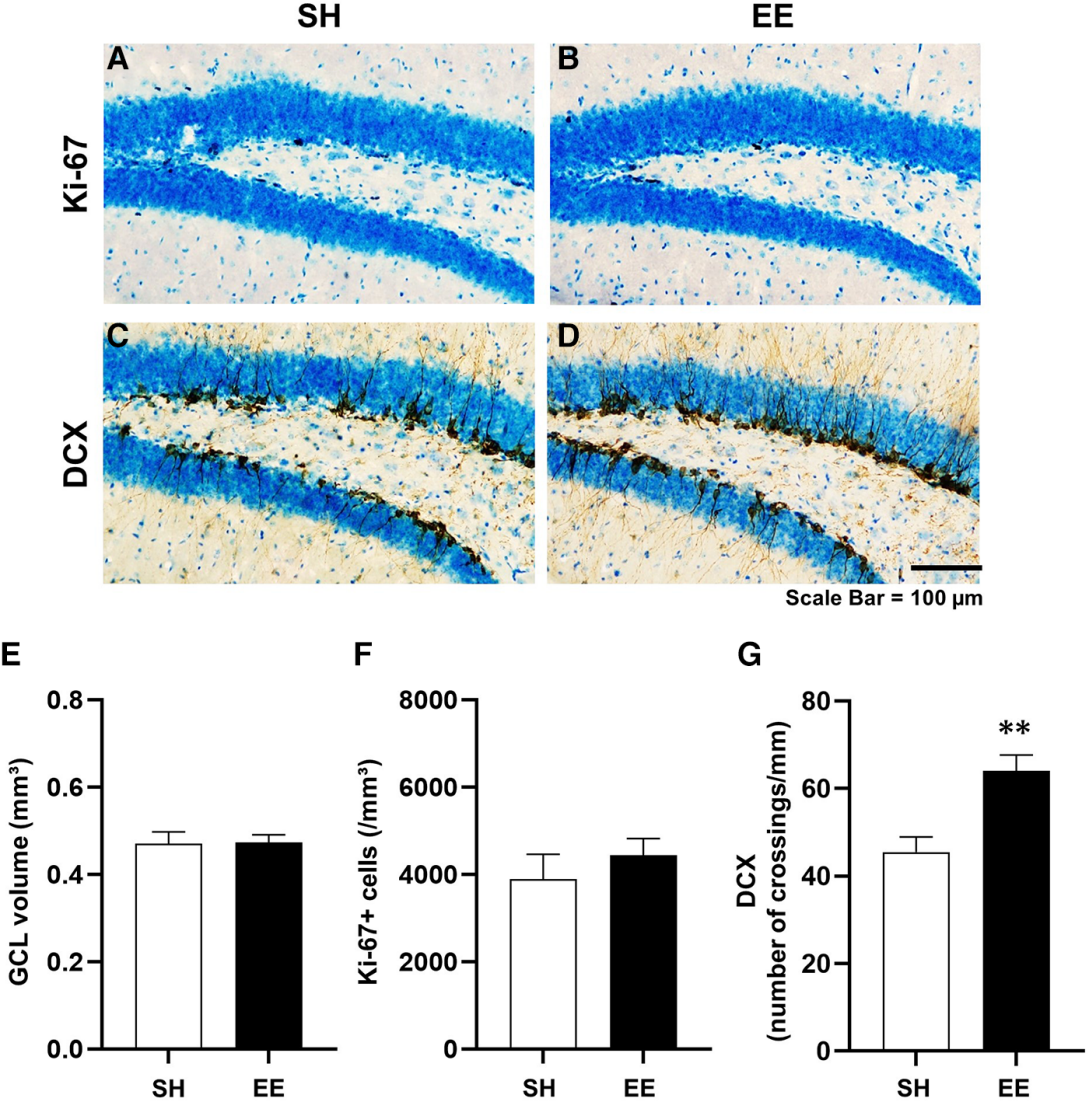
Representative images showing Ki-67-positive cells (**A,B**) and doublecortin-positive immature neurons (**C,D**) in the hippocampal dentate gyrus. Estimated volume of the granule cell layer of the hippocampal dentate gyrus (**E**), densities of Ki-67-positive cells (**F**), and DCX-positive immature neurons (**G**) **P < 0.01 vs. SH. Values represent means ± SEM (*n* = 4–6 per group).

We assessed hippocampal neurogenesis in the SH and EE mice at the end of the experiment. Representative images of immunohistochemical staining are shown in Figures 3A–D. The densities of DCX-positive immature neurons were significantly higher in the EE mice than in the SH mice [F(7,3) = 2.218, *P* < 0.01], indicating that hippocampal neurogenesis was enhanced in the EE mice. Although there were no significant differences between the groups in the estimated total volume of the GCL [F(3,7) = 1.28, *P* = 0.905] and the densities of Ki-67-positive cells [F(3,7) = 1.09, *P* = 0.437], the result of the DCX suggests that our environmental intervention provided enriched housing conditions for the mice.

## 4. Discussion

We demonstrated that an enlarged housing space and increased spatial complexity caused by the placement of a variety of objects did not increase physical activity in mice. In contrast, hippocampal neurogenesis was enhanced in the EE mice, indicating that environmental interventions provided sufficiently enriched housing conditions for these mice. These findings suggest that physical activity levels may be determined independently of housing spaces and environmental spatial complexity.

To clarify the simple effect of enlarging housing space on physical activity, we employed an enrichment procedure in which the housing space was first gradually enlarged, and then a variety of objects were placed in the cage to examine the specific effect of each enrichment component on physical activity in mice (Figure 1). Although the materials of the cages were different (polycarbonate for the standard and large cages, and polypropylene for the extra-large cage), this would not affect the physical activity level because no differences were found between the large cage and the extra-large cage. On the first day of each housing condition, physical activity in EE mice was transiently increased, presumably caused by environmental novelty (13). Importantly, the physical activity levels were comparable between groups after the second day of each condition. These results indicate that the enlargement of housing space did not increase physical activity in the mice (Figure 2). However, this finding is inconsistent with previous research demonstrating that a large cage is associated with higher physical activity levels (19, 20). Although the above-mentioned previous studies have measured physical activity in mice using a force plate or a gravimetric method, these methods are unable to assess individual physical activity levels in group-housed rodents. Due to this limitation, caution should be exercised in drawing conclusions about the effect of enlarged housing space on physical activity. Considering this, our finding that enlarged housing spaces did not increase physical activity may significantly improve the understanding of the regulation of physical activity in response to enlarged housing spaces.

Mice in the EE group were finally housed in the extra-large cage with a variety of objects for three weeks. The placement of a variety of objects increases spatial complexity in the cage, and objects with different materials may affect the mice's perception. However, the addition of a variety of objects did not increase physical activity in the mice, indicating that increased spatial complexity was incapable of increasing physical activity (Figure 2). To date, physical activity in rodents has not been assessed in a complex environment, including those with a variety of objects, using existing devices for the measurement of physical activity, such as infrared, telemetry, and video-tracking systems (13). To the best of our knowledge, this is the first study to demonstrate the effects of environmental spatial complexity on physical activity in mice. Surprisingly, physical activity was unchanged depending on our enrichment procedure, even though the housing environments of SH and EE mice were obviously different, and EE mice exhibited enhanced hippocampal neurogenesis. These findings may indicate that providing only spatial opportunities, including areas of locomotion and a variety of objects, is insufficient to promote physical activity and may suggest the importance of interventions closely related to physical activity behavior or requiring locomotion.

In the current study, EE did not stimulate cell proliferation in the hippocampus. However, we found that the density of DCX-positive immature neurons was increased in the EE mice (Figure 3), indicating that EE enhanced hippocampal neurogenesis. This result shows that our environmental intervention successfully provided enriched housing conditions for the mice. It is interesting to note that hippocampal neurogenesis was enhanced without increasing physical activity. We do not consider that this unexpected result suggests that physical activity is not associated with enhanced hippocampal neurogenesis, and a more careful discussion is needed. Physical activity in an environment with increased spatial complexity is likely to be contextually different from physical activity in the standard cage. Assuming that opportunities for spatial recognition and learning increase in a spatially complex environment, the context of physical activity differs between SH and EE mice, even though the activity volume was not different. In the previous human study, the effectiveness of a 60-minute walk in a natural environment compared to an urban environment on decreasing neural activity in stress-related brain regions was demonstrated (21), suggesting that contexts of physical activity related to environmental settings and locations are potentially important for the benefit of physical activity. Based on recent literature showing that contextual factors, such as location and type of activity, can affect the impact of exercise on mood (22), our findings suggest that the environment in which physical activity is performed could be an important contextual factor, as well as the amount of physical activity, which influences the effects of physical activity on hippocampal function. As a result, it may be feasible to consider not only the volume of physical activity but also the context of physical activity as a new assessment of activity conditions in physical activity guidelines.

## 5. Conclusion

Our study revealed that providing mice with larger housing spaces and increased spatial complexity did not increase their physical activity, which sheds light on the response of physical activity to environmental changes. Further investigations are required to examine the factors that can promote physical activity. In contrast, we also found that physical activity in an environment with higher spatial complexity improved hippocampal neurogenesis without a corresponding increase in activity volume. This highlights the potential influence of the activity environment as an essential contextual factor in enhancing hippocampal function through physical activity.

## Data Availability

The raw data supporting the conclusions of this article will be made available by the authors, without undue reservation.
